# Integrated analysis to identify the AC005154.6/hsa-miR-29c-3p/CCNL2 axis as a novel prognostic biomarker associated with immune infiltration in prostate cancer

**DOI:** 10.1186/s12935-022-02779-5

**Published:** 2022-11-11

**Authors:** Qinyu Li, Bingliang Chen, Guoda Song, Kai Zeng, Xin Chen, Jianping Miao, Xianglin Yuan, Jihong Liu, Zhihua Wang, Bo Liu

**Affiliations:** 1 Department of Urology, Tongji Hospital, Tongji Medical College, Huazhong University of Science and Technology, Wuhan, 430030 Hubei China; 2 Department of Oncology, Tongji Hospital, Tongji Medical College, Huazhong University of Science and Technology, Wuhan, 430030 Hubei China; 3 Department of Geriatrics, Tongji Hospital, Tongji Medical College, Huazhong University of Science and Technology, 430030, Wuhan, Hubei China

**Keywords:** Competing endogenous RNAs, CCNL2, Immune infiltration, Methylation, Prostate cancer

## Abstract

**Background:**

Prostate cancer (PCa) is currently the major malignancy in men. It is becoming increasingly clear that competitive endogenous RNA (ceRNA) regulation networks are important in a wide variety of cancers. Nevertheless, there is still much to learn about the biological functions of the ceRNA network in prostate cancer.

**Methods:**

The ceRNA network was constructed using the "GDCRNATools" package. Based on survival analysis, we obtained AC005154.6/hsa-miR-29c-3p/CCNL2 for further analysis. The prognostic model based on this ceRNA network was constructed by univariate and multivariate Cox regression methods. Furthermore, functional enrichment analysis, mutation landscape analysis, immune infiltration analysis, drug sensitivity analysis, methylation analysis, pan-cancer analysis, and molecular experiments of CCNL2 were carried out to investigate the role of CCNL2 in tumorigenesis.

**Results:**

We identified the AC005154.6/CCNL2 axis as a risk factor that can promote the progression of prostate cancer by bioinformatics analysis and molecular experiments. Immune infiltration analysis suggested that CCNL2 may act as a novel biomarker for treatment decisions. The methylation level of CCNL2 was significantly decreased in tumor samples, possibly contributing to the upregulation of CCNL2 in prostate cancer. Moreover, CCNL2 is differentially expressed in multiple cancers and is tightly correlated with immune infiltration.

**Conclusion:**

The current study constructed a ceRNA network, AC005154.6/hsa-miR-29c-3p/CCNL2. Potentially, this biomarker can be used for early diagnosis and decision-making about prostate cancer treatment.

**Supplementary Information:**

The online version contains supplementary material available at 10.1186/s12935-022-02779-5.

## Introduction

There is no doubt that prostate cancer (PCa) is one of the most common types of cancer among men [1]. Among the newly diagnosed cases of prostate cancer, nearly eighty percent are localized. However, there are still many cases of advanced or metastatic disease [2]. Although many localized prostate cancers have a good prognosis, a significant decrease in survival probability occurs in castration-resistant prostate cancer (CRPC). In advanced cases, survival rates range from 26 to 30% [3]. Nevertheless, therapeutic options are still limited for these patients [4].

Prostate cancers that are advanced, metastatic, or recurrent are treated with androgen deprivation therapy (ADT). However, ADT resistance occurs in some patients, resulting in a lack of effective treatment options and a poor prognosis [5]. On that account, immunotherapy has become an important option for advanced prostate cancer, especially for CRPC [6]. In fact, patients with durable responses to immune checkpoint inhibitors showed a marvelous survival benefit. Unfortunately, for the majority of patients, immune checkpoint blockade provides minimal or no clinical benefit. Therefore, reliable validated biomarkers of ADT resistance are necessary for timely treatment correction. Moreover, biomarkers that can predict immunotherapy response or enhance the results of immunotherapy might revolutionize the treatment of prostate cancer.

Long non-coding RNAs (lncRNAs) are a subtype of ncRNAs that are longer than 200 nt and lack the ability to encode proteins [7]. Previous studies have revealed that lncRNAs have a critical impact on tumor progression [8–10]. microRNA (miRNA) is a small RNA that binds to the 3' untranslated region of its target mRNA to promote gene silencing [11, 12]. It has previously been observed that miRNAs are associated with the ADT response and CRPC development through several mechanisms [5].

Salmena et al. proposed competitive endogenous RNA (ceRNA) as a mechanism to regulate gene expression in 2011 [13]. The hypothesis thought that miRNA target sites can be competitively bound by endogenous RNA. Therefore, indirect regulation of miRNA target genes can be achieved by endogenous RNAs [14], which is also called the miRNA sponge effect. It has been reported that the ceRNA network may play crucial roles in the development of different cancers, such as gastric cancer [15], colorectal cancer [16], hepatocellular carcinoma [17] and lung adenocarcinoma [18]. However, whether ceRNA networks play critical roles in the progression of prostate cancer still needs further research.

In this study, we first identified DEmRNAs, DEmiRNAs, and DElncRNAs from the TCGA and GEO databases. Then, we constructed ceRNA networks by the “GDCRNATools” package. Based on survival analysis, ceRNA networks which may play crucial roles in prognosis in PRAD patients were identified. Among them, AC005154.6/hsa-miR-29c-3p/CCNL2 showed the highest correlation between lncRNA and mRNA, and was selected for further analysis. We constructed a three-gene-based prognostic model based on this ceRNA network and compared its efficiency with the model that consisted of the clinicopathological features. Furthermore, Multi‐Omics analysis and molecular experiments were applied to investigate the potential biological function of the AC005154.6/hsa-miR-29c-3p/CCNL2 axis in PCa. This ceRNA network may help us to better comprehend the pathogenesis of PCa and provide a novel biomarker for early diagnosis and treatment decisions in prostate cancer.

## Materials and methods

### Data acquisition and processing

RNA-Seq data, including mRNA, lncRNA and miRNA expression data of prostate cancer patients, somatic mutation and clinical information from the TCGA database and the GEO database (GSE46602 [19], GSE6956 [20], GSE70768 [21]), were downloaded. All raw data were preprocessed in R software.

### Screening of differentially expressed genes

For data from the TCGA database, we identified DEmRNAs, DEmiRNAs, and DElncRNAs by the “edgeR” package [22]. The DEmRNAs and DElncRNAs were selected with |logFC|> 0.5 and FDR < 0.05, and the DEmiRNAs were chosen with |logFC|> 0.3 and FDR < 0.05. For data from the GEO database, DEmRNAs were identified with |logFC|> 1 and FDR < 0.05 by the “limma” package [23]. Furthermore, DEmRNAs, which existed in both TCGA DEmRNAs and the union of DEmRNAs from three GEO series, DEmiRNAs and DElncRNAs were selected to construct ceRNA networks.

### CeRNA network construction and survival analysis

Prediction of mRNA‒miRNA and lncRNA‒miRNA pairs was performed using the "GDCRNATools" package [24]. To identify competing endogenous interactions, three criteria were used (Additional file 2). The mRNAs, miRNAs, and lncRNAs that were associated with progression-free survival (PFS) were identified using univariate Cox regression. For further analysis, we selected the networks in which mRNAs, miRNAs and lncRNAs were all associated with prognosis. The network with the highest correlation between mRNA and lncRNA was selected for further analysis. Based on starBase v2.0 and TargetScan, potential miRNA‒lncRNA and miRNA‒mRNA target sites were predicted.

### Construction of the prognostic model

The riskscore based on the expression of AC005154.6, hsa-miR-29c-3p and CCNL2 was estimated by the following formula: riskscore = (β1 * RNA1 expression level) + (β2 * RNA2 expression level) + (β3 * RNA3 expression level). The cut-off point was determined using the “survminer” package [25]. Subsequently, Kaplan‒Meier survival curves were applied to estimate the ability of the model to distinguish different subtypes of patients. The efficiency of the model was evaluated by time-dependent ROC curves.

### Relationship between the prognostic model and clinical parameters

The riskscore was compared between patients with different clinical parameters to determine if it correlates with cancer progression. Univariate and multivariate Cox regression analyses were carried out to determine whether the prognostic value of the model changed when combining clinical parameters. Furthermore, by using the R package “regplot” [26], a nomogram was visualized based on multivariate Cox regression, and calibration curves were drawn to indicate its accuracy. The C-index of the model proposed by us was estimated and compared with the C-index of the clinical parameter model, which consists of the Gleason score, T stage, N stage and M stage.

### Mutation landscape analysis and functional enrichment analysis of CCNL2

We downloaded somatic mutation data from the TCGA database and selected "mutect2" data for further analysis. The mutation landscape between the CCNL2^high^ and CCNL2^low^ groups was visualized. To understand the potential biological processes and pathways in which CCNL2 is involved, GeneMANIA was applied to identify the functionally similar genes of CCNL2, and GSEA was conducted by the “clusterprofiler” package [29].

### The association between immune infiltration levels and CCNL2

To explore the relationship between CCNL2 expression and infiltrating immune cells, we utilized TIMER [30] to explore its correlations with the infiltration levels of immune cells. Moreover, a correlation analysis between immune checkpoint gene expression and the ceRNA network was performed. Given that CD8 + T cells targeting tumor cells play vital roles in tumor immunity, we tested whether CCNL2 expression correlated with CD8 + T cell infiltration. The microsatellite instability (MSI) score helps to identify patients who could benefit from immune checkpoint inhibitors [31], and its association with CCNL2 expression was estimated.

### Drug sensitivity analysis

For the investigation of candidate small molecules for PCa treatment, the CMap database was used. Differentially expressed genes between the CCNL2^high^ and CCNL2^low^ groups were uploaded, and we performed initial identification of potential drug interventions. We also compared the susceptibility to bicalutamide and docetaxel across the CCNL2^high^ and CCNL2^low^ groups using the GDSC database by applying the pRRophetic [32] package. Additionally, RNA-seq data from CellMiner were downloaded. FDA-approved or clinically trial drugs were analysed. The expression of CCNL2 and drug susceptibility have been correlated.

### Methylation analysis of CCNL2

Previous studies have found that DNA methylation is an important epigenetic mechanism for oncogenesis. The expression levels of 3 DNA methyltransferases (DNMT1, DNMT3A, and DNMT3B) which enable to regulate gene expression and have an impact on the characteristics of tumors were compared in the CCNL2^high^ and CCNL2^low^ groups. We applied UALCAN [33] and DiseaseMeth version 2.0 to assess the methylation levels of CCNL2 between PCa and normal tissues. Moreover, we analysed the correlation of CCNL2 expression and its DNA methylation state by MEXPRESS.

### Pan-cancer analysis of CCNL2

The differential expression of 33 cancer types was examined using TIMER. TCGA RNA-seq data were downloaded to assess the correlation between CCNL2 expression and immune cell infiltration in 33 cancer types. Univariate Cox regression analysis was used to determine if there was a potential relationship between CCNL2 expression and OS and PFS of 33 cancer types.

### Validation of CCNL2 in molecular experiments

Four paired PCa and matched peritumor tissues were collected at Tongji Hospital. This research was approved by the Tongji Hospital Ethics Committee. The CWR22Rv1 cell line was purchased from Shanghai Institute of Cell Biology (Shanghai, China), and C4–2b cells were obtained from Wuhan Shanen Biotechnology (Wuhan, China). The cell lines were cultured in RPMI1640 medium with 10% fetal bovine serum. In Additional file 3: Table S1, siRNA sequences for C4-2b and CWR22Rv1 cells are listed. Protein samples were extracted from four paired tumor-adjacent tissues and siRNA-transfected cells. Total proteins were loaded onto SDS‒PAGE gels, separated by electrophoresis, and transferred to PVDF membranes. Antibodies were then incubated on the membranes (CCNL2: A14938, Wuhan ABclonal Technology; GAPDH: A00227-1, Wuhan Boster Biological Technology).

To explore the role of CCNL2 in prostate cancer, a scratch assay was executed by making a straight longitudinal incision with a pipette tip on the monolayer of cells. The scratch widths were then estimated to reveal the migration and invasion of cells after 48 h. We performed a colony formation assay. After siRNA transfection, cells were trypsinized at 24 h, seeded into 6-well plates (2 × 10^4^/well) and grown for up to 2 weeks. Moreover, cell proliferation was also analysed by CCK8 assay.

### Statistical analysis

All the data were analysed and visualized by using R software 4.0.4 and GraphPad Prism 8.0. For comparisons between two groups, Student's t test and Wilcoxon test were applied. Correlation analysis was performed by Spearman correlation test. P values < 0.05 were regarded as statistically significant.

## Results

### Identification of DEmRNAs, DEmiRNAs and DElncRNAs in PRAD

**Fig. 1:**
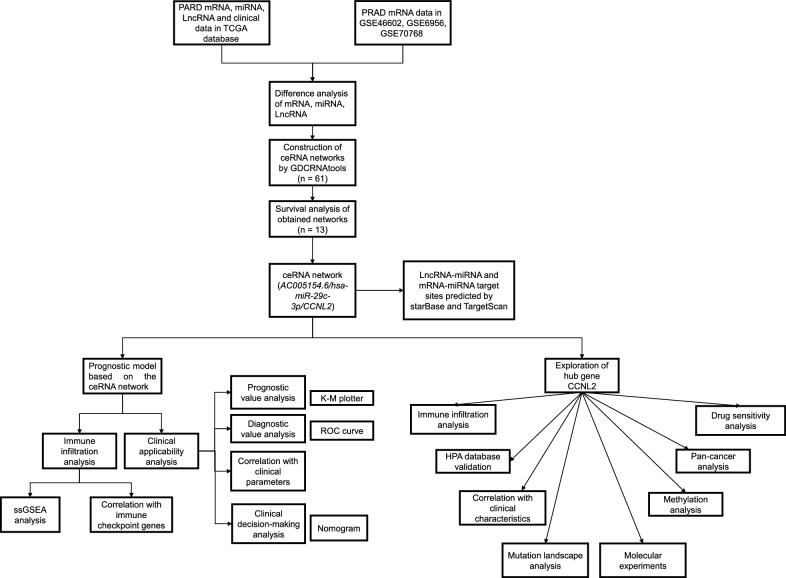
Flow diagram of the construction and analysis of ceRNA networks

**Fig. 2:**
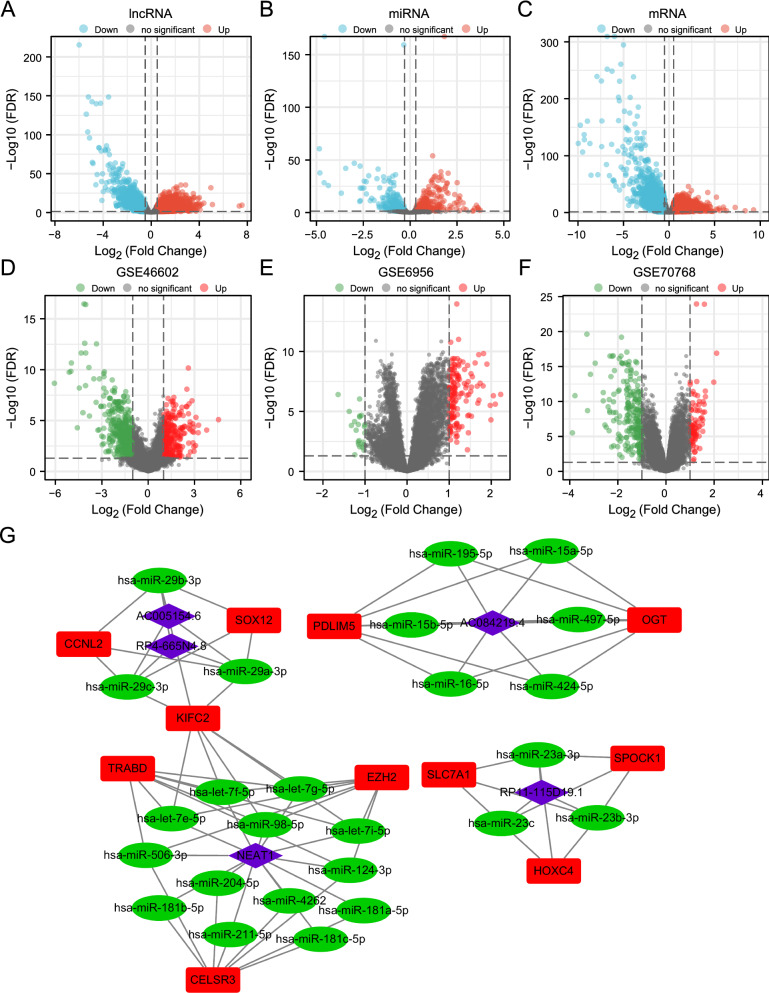
Volcano plots of DElncRNAs, DEmiRNAs, DEmRNAs and construction of ceRNA: **A**–**C** The volcano plots of DElncRNAs, DEmiRNAs, DEmRNAs for the TCGA data. **D**–**F** The volcano plots of DEmRNAs in three GEO series: GSE46602, GSE6956, and GSE70768. **G** Construction of the ceRNA networks. The purple diamonds represent lncRNAs. The green ellipses represent miRNAs. The red rectangles represent mRNAs

Figure 1 shows the research roadmap. After processing the raw data from TCGA, we identified 6559 DEmRNAs, 485 DEmiRNAs, and 4490 DElncRNAs in PRAD (Fig. 2A-C). Three GEO series that compared prostate cancer cells and benign prostate glands (GSE46602), prostate tumor tissue and surrounding normal prostate tissue (GSE6956), and castration-resistant prostate cancer and prostate tumor tissues (GSE70768) were applied for the identification of differentially expressed genes in the progression of prostate cancer. We identified 891 DEmRNAs in GSE46602, 162 DEmRNAs in GSE6956 and 309 DEmRNAs in GSE70768 (Fig. 2D-F). We intersected DEmRNAs from the TCGA database and the union of DEmRNAs discovered by these three GEO series to analyze further.

### Identification of the AC005154.6/hsa-miR-29c-3p/CCNL2 axis

The identified DEmRNAs, DEmiRNAs, and DElncRNAs were used to build the ceRNA network. There were 61 ceRNA networks identified as differentially expressed profiles (Additional file 4: Table S2). Through survival analysis, we discovered 8 miRNAs (Additional file 1: Figure S1), 19 mRNAs (Additional file 1: Figure S2) and 9 lncRNAs (Additional file 1: Figure S3) that were associated with the prognosis of prostate cancer. The survival analysis revealed 13 ceRNA networks (Fig. 2G) (Additional file 5: Table S3). Among them, AC005154.6/hsa-miR-29c-3p, hsa-miR-29b-3p, and hsa-miR-29a-3p/CCNL2 showed the highest correlation between mRNA and lncRNA (coefficient of correlation is 0.8) (Additional file 1: Figure S4) and have not previously been reported in PRAD. Based on this, we selected this ceRNA network for further analysis.

**Fig. 3:**
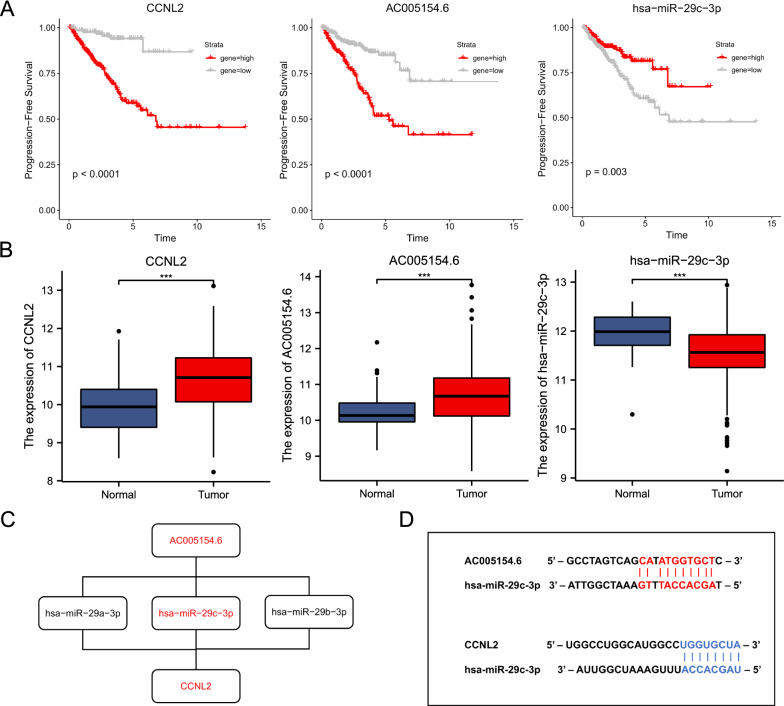
Progression-free survival analysis and differential expression analysis of the AC005154.6/hsa-miR-29c-3p/CCNL2 axis. **A** The high- and low-expression of AC005154.6, hsa-miR-29c-3p and CCNL2 were compared by a Kaplan‒Meier survival curve. **B** The expression patterns of AC005154.6, hsa-miR-29c-3p and CCNL2 between tumor and adjacent normal tissues. **C** Schematic model of the AC005154.6/hsa-miR-29c-3p/CCNL2 axis. **D** The potential target sites of miRNA‒mRNA (blue) and miRNA‒lncRNA (red) predicted by starBase v2.0 and TargetScan. (*** p < 0.001)

Survival analysis showed that AC005154.6, hsa-miR-29c-3p and CCNL2 were associated with the prognosis of PCa (Fig. 3A). The expression of AC005154.6 and CCNL2 was upregulated in PCa, while the expression of hsa-miR-29c-3p was decreased (Fig. 3B). These data indicated that AC005154.6 may upregulate the expression of CCNL2 by sponging hsa-miR-29c-3p. Thus, the AC005154.6/hsa-miR-29c-3p/CCNL2 ceRNA network was found (Fig. 3C). Furthermore, by using starBase and TargetScan, we predicted the target sites in the AC005154.6 and CCNL2 3’UTRs paired with hsa-miR-29c-3p (Fig. 3D).

### Establishment of a ceRNA-based prognostic model

To explore the prognostic value of the AC005154.6/hsa-miR-29c-3p/CCNL2 network, we assessed the role of this axis in the PFS of PRAD patients (Additional file 6: Table S4). Patients were split into high- and low-risk groups based on the cut-off obtained from the “survminer” package (Additional file 1: Figure S5). Kaplan‒Meier curves revealed that patients with high riskscore suffered poor clinical outcomes (Additional file 1: Figure S5). According to the outcome of time-dependent ROC analysis, the AUC was 0.684 at 1 year, 0.691 at 2 years, 0.717 at 3 years, 0.673 at 4 years and 0.664 at 5 years (Additional file 1: Figure S6).

### Association between clinical characteristics and the riskscore

**Fig. 4:**
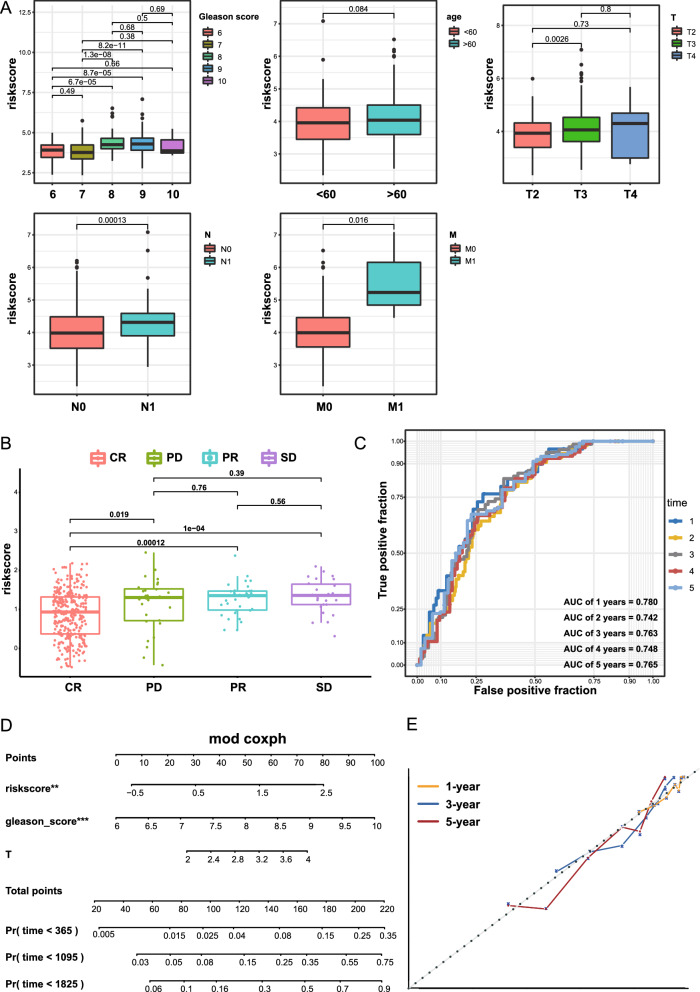
The correlation of the riskscore with clinical parameters. **A** Comparison of riskscore among different Gleason scores, ages, T stages, N stages, and M stages. **B** The riskscore in patients with different primary treatment outcomes. **C** Time-dependent ROC curve of the nomogram. **D** A nomogram based on 3 variables, including the riskscore, Gleason score and T stage, was developed for the prediction of the 1-, 3-, and 5-year progression-free survival in PRAD. **E** Calibration plot of the nomogram

To estimate the clinical independence of the prognostic model, we applied Cox regression analysis. According to the univariate Cox regression, the riskscore was a risk factor for survival (HR = 2.72, p value < 0.001) (Additional file 1: Figure S7). The results of multivariate Cox regression further confirmed the significance of the prognostic model (HR = 1.84, p value = 0.003) (Additional file 1: Figure S7). Moreover, with the progression of the tumor, the riskscore was also elevated (Fig. 4A). The riskscore of complete response (CR) was obviously lower than other outcomes (Fig. 4B), and the high-risk group had a higher proportion of stable disease (SD) and progressive disease (PD) (Additional file 1: Figure S8). Finally, a nomogram including factors with a p value < 0.05 in multivariate Cox regression analysis (Gleason score, T stage and riskscore) was developed (Fig. 4D). There was a good predictive value for the nomogram based on calibration plots (Fig. 4E). After combining clinical parameters, the AUC was 0.780 at 1 year, 0.742 at 2 years, 0.763 at 3 years, 0.748 at 4 years and 0.765 at 5 years (Fig. 4C). Thereafter, we calculated the C-index of the model proposed by us and compared it with the model which consists of the Gleason score, T stage, N stage and M stage (Table 1). The results, 0.733 for our model and 0.711 for the clinical parameters model, indicated that the combination of riskscore with clinical parameters may enhance the diagnostic accuracy.

**Table 1:** Comparison of the nomogram and model consisting of clinical parameters

Variable	Nomogram			Clinical parameters		
	β	Odds Ratio	P value	β	Odds Ratio	P value
Gleason_score	0.584	1.794	< 0.001	0.714	2.043	< 0.001
T stage	0.436	1.547	0.088	0.530	1.699	0.042
N stage	NA	NA	NA	−14.824	< 0.001	0.995
M stage	NA	NA	NA	−0.260	0.771	0.340
Riskscore	0.552	1.736	0.005	NA	NA	NA
C-index	0.733			0.711		

### High expression of CCNL2 indicates poor prognosis in prostate cancer

**Fig. 5:**
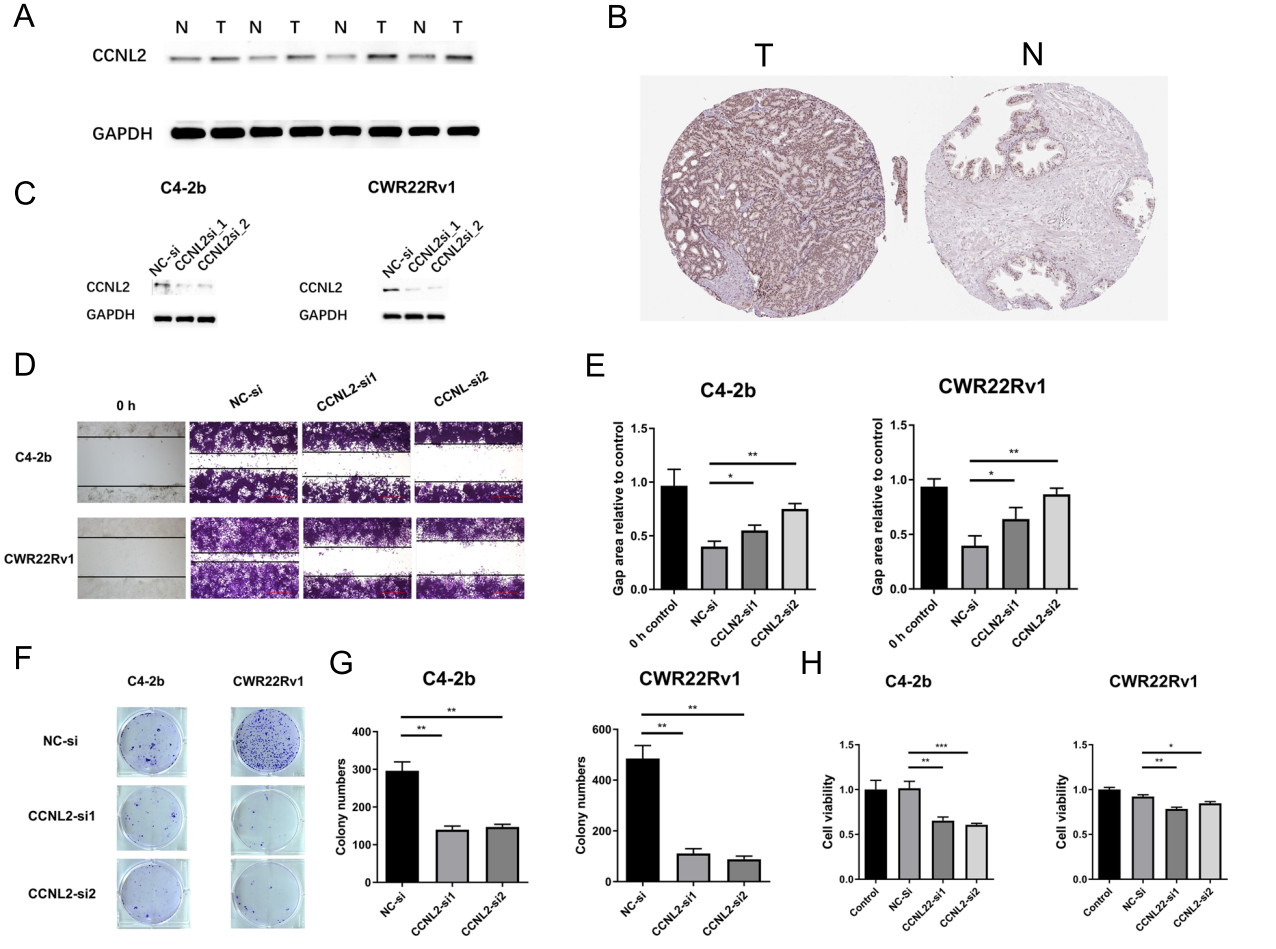
Validation of CCNL2 in molecular experiments: **A** CCNL2 expression between tumor and paired normal tissue analysed by western blot. **B** Immunohistochemical staining for CCNL2 in HPA. **C** The expression of CCNL2 in C4-2b and CWR22Rv1 cells after siRNA transfection. **D**–**E** Scratch assay in C4-2b and CWR22Rv1 cells. **F**–**G** Colony formation in C4-2b and CWR22Rv1 cells. **H** Cell counting kit-8 of C4-2b and CWR22Rv1 after 48 h. (* p < 0.05, ** p < 0.01, *** p < 0.001)

To better comprehend the role of the AC005154.6/hsa-miR-29c-3p/CCNL2 axis in PRAD, we next mainly concentrated on the characteristics of CCNL2. We used UALCAN to uncover the clinical relevance of CCNL2 expression, which showed a positive association between sample type and nodal metastasis status (Additional file 1: Figure S9). To further validate the upregulation of CCNL2 in PCa, the protein expression in four pairs of tumors and peritumor tissues was analysed by western blotting. The results indicated that CCNL2 expression was significantly increased in PCa (Fig. 5A). Moreover, the HPA database substantiated that CCNL2 was positive in PCa tissue and nearly negative in normal tissue (Fig. 5B). Subsequently, siRNA transfection targeting CCNL2 was conducted, and CCNL2 expression significantly decreased in both C4–2b and CWR22Rv1 cells (Fig. 5C). Downregulation of CCNL2 suppressed tumor cell migration (Fig. 5D–E) and colony formation (Fig. 5F–G). Finally, the results of CCK8 also revealed that cell proliferation was restrained when CCNL2 was downregulated (Fig. 5H). All the results indicated that prostate cancer proliferation and migration may be promoted by CCNL2.

### Mutation landscape analysis and functional enrichment analysis of CCNL2

The mutation landscape of genes with high mutation frequencies in CCNL2^high^ and CCNL2^low^ is presented (Additional file 1: Figure S10). CCNL2^high^ group was found to contain a greater number of mutation types and more mutated samples. Moreover, functional enrichment analysis was performed. The results of GeneMANIA revealed that the functions of CCNL2 and its most correlated genes were primarily related to the regulation of cyclin-dependent protein kinase activity, protein kinase regulator activity and kinase regulator activity (Additional file 1: Figure S11). The GSEA results indicated that the upregulation of CCNL2 was associated with pathways such as salmonella infection, proteoglycans in cancer and dilated cardiomyopathy.

### CCNL2 as a potential biomarker to predict therapeutic benefits

**Fig. 6:**
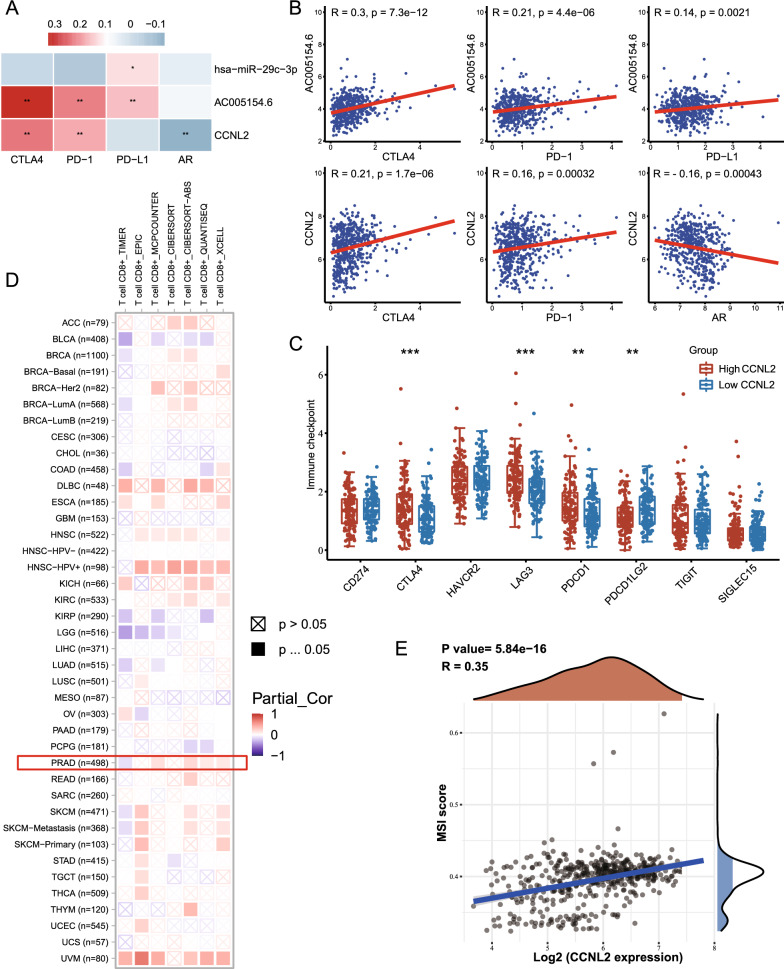
The correlation between the expression of CCNL2 and the efficacy of immunotherapy: **A**–**B** Association between the expression of AC005154.6, hsa-miR-29c-3p, and CCNL2 and the expression of immune checkpoint genes and androgen receptor. **C** The expression of immune checkpoint genes in the CCNL2^high^ and CCNL2^low^ groups. **D** The correlation of CCNL2 expression and the infiltration levels of CD8 + T cells. **E** Correlation analysis between CCNL2 expression and MSI score. (** p < 0.01, *** p < 0.001)

Based on the results in TIMRE, the infiltration levels of the majority of immune cells are associated with altered CCNL2 gene copy numbers (Additional file 1: Figure S12). To better guide clinical medication, we evaluated the correlation of immune checkpoint genes and AR with the ceRNA network (Fig. 6A–C). CTLA4, PD-1, PD-L2, LAG3 and AR are closely connected with CCNL2, suggesting the potentiality for CCNL2 to serve as a biomarker in predicting patient response to ADT and immunotherapy. CD8 + T cells play an essential role in the clinical activity of immunotherapy. We assessed the correlation of CCNL2 expression and CD8 + T cell infiltration levels in 33 types of cancer. The results showed a positive correlation between CCNL2 expression and CD8 + T cell infiltration levels by multiple algorithms (Fig. 6D). Moreover, we discovered that the MSI score was positively correlated with CCNL2 expression in PRAD (Fig. 6E). All the results suggested that patients with high levels of CCNL2 may benefit from immunotherapy.

**Fig. 7:**
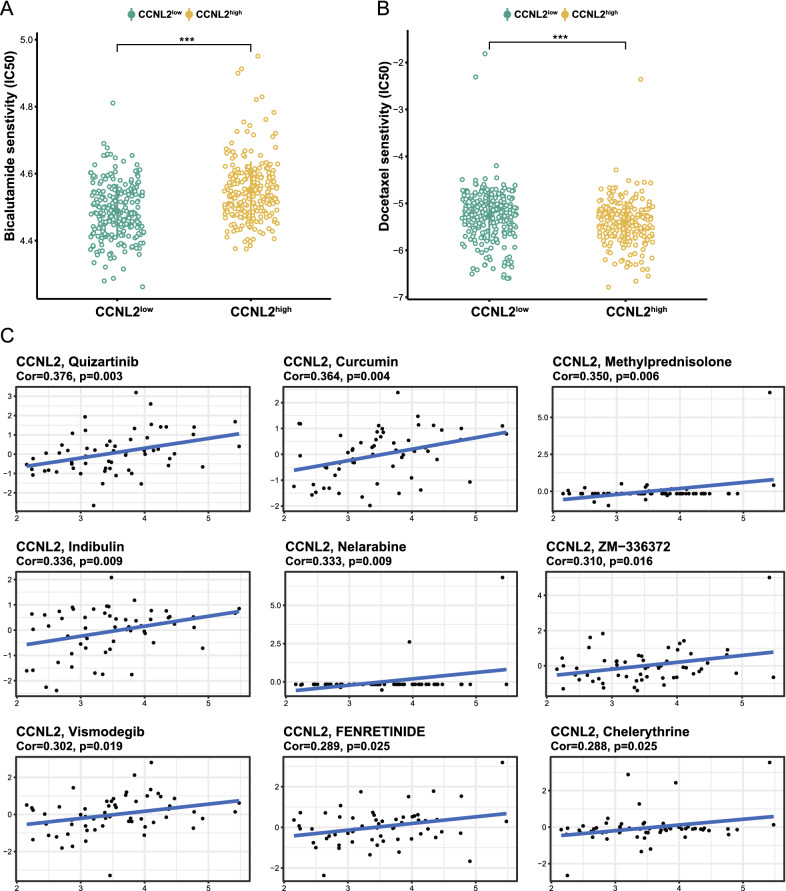
Drug sensitivity analysis: **A**–**B** Predicted IC50 values of bicalutamide and docetaxel between the CCNL2^high^ and CCNL2^low^ groups. **C** The correlations of sensitivity to anticancer drugs and CCNL2 expression assessed by CellMiner

Given that the treatment of prostate cancer still relies heavily on ADT and chemotherapy. Correlations of drug sensitivity and CCNL2 expression were explored using the GDSC database. As expected, the results revealed that patients with high expression of CCNL2 were not sensitive to bicalutamide (Fig. 7A). Nevertheless, these patients seemed to be sensitive to docetaxel (Fig. 7B). Through the CellMiner database, a significant association was found between CCNL2 expression and 22 drug sensitivities (Additional file 7: Table S5), and the top nine drugs are shown in Fig. 7C. Moreover, we explored CMap to identify anti-PCa micromolecular drugs based on the differentially expressed genes between CCNL2^high^ and CCNL2^low^ patients. Twenty-nine small molecules were identified as possible drugs to treat PCa (Additional file 8: Table S6).

### Methylation and pan-cancer analysis of CCNL2

**Fig. 8:**
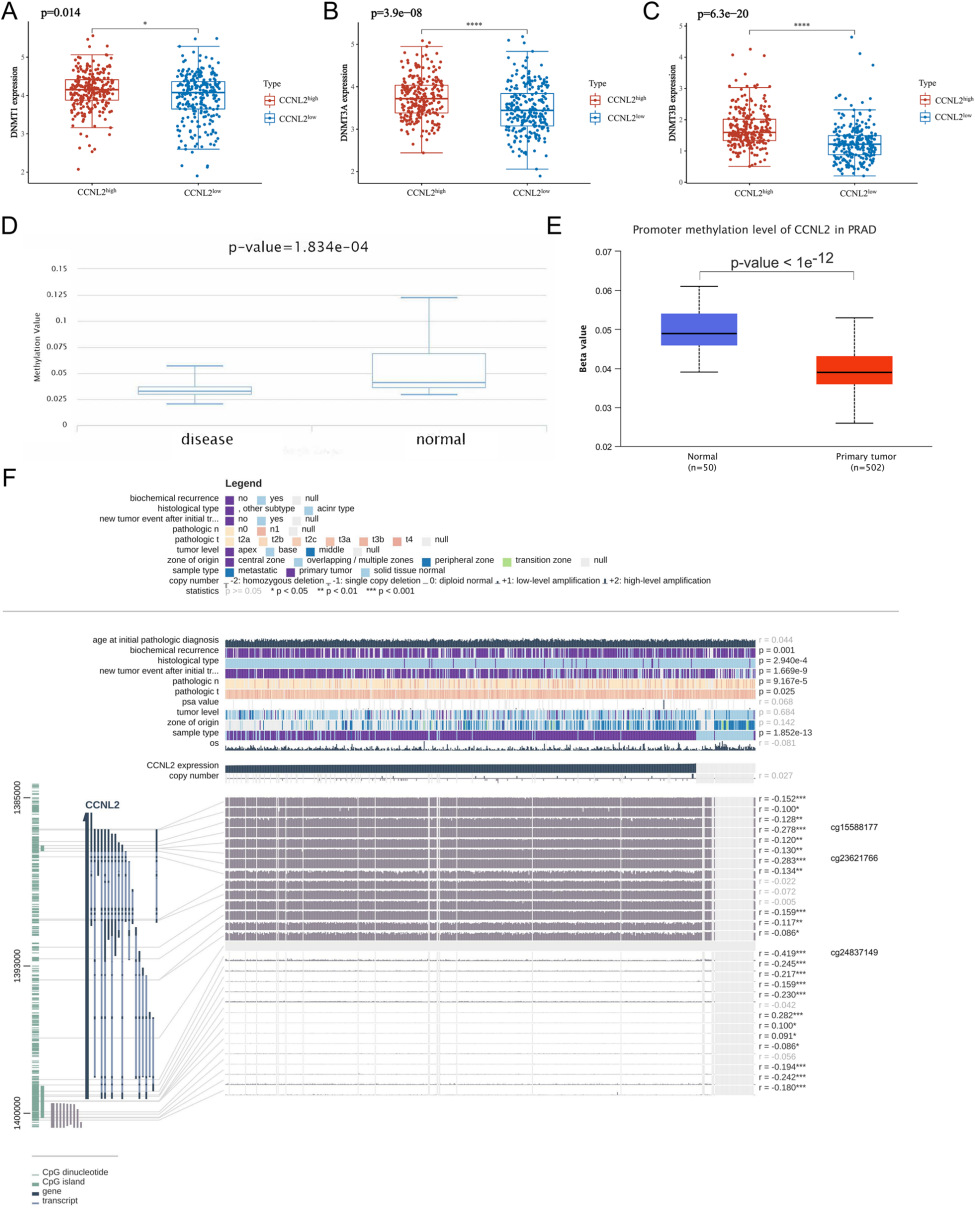
Methylation analysis of CCNL2: **A**–**C** Differential expression of three DNA methyltransferases (DNMT1, DNMT3A, and DNMT3B). **D** Methylation of CCNL2 evaluated by using DiseaseMeth version 2.0. **E** Methylation of CCNL2 assessed by UALCAN. **F** The methylation site of the CCNL2 DNA sequence associated with gene expression was visualized using MEXPRESS. (* p < 0.05, ** p < 0.01, *** p < 0.001, **** p < 0.0001)

To further elucidate the mechanisms of abnormal upregulation of CCNL2 in PCa, the correlation between CCNL2 expression and its methylation status was estimated. The expression of three DNA methyltransferases in the CCNL2^high^ and CCNL2^low^ groups was compared, and the results suggested that the expression of all three methyltransferases was markedly increased in the CCNL2^high^ group (Fig. 8A–C). Additionally, DiseaseMeth version 2.0 suggested that the methylation level of CCNL2 was significantly lower in PCa tissues (Fig. 8D). Similarly, the results of UALCAN also suggested that the methylation level was markedly decreased in PCa tissues (Fig. 8E). Furthermore, we discovered that most methylation sites of CCNL2 were negatively associated with their expression levels, and the three most significant sites were cg24837149, cg23621766 and cg15588177 (Fig. 8F).

**Fig. 9:**
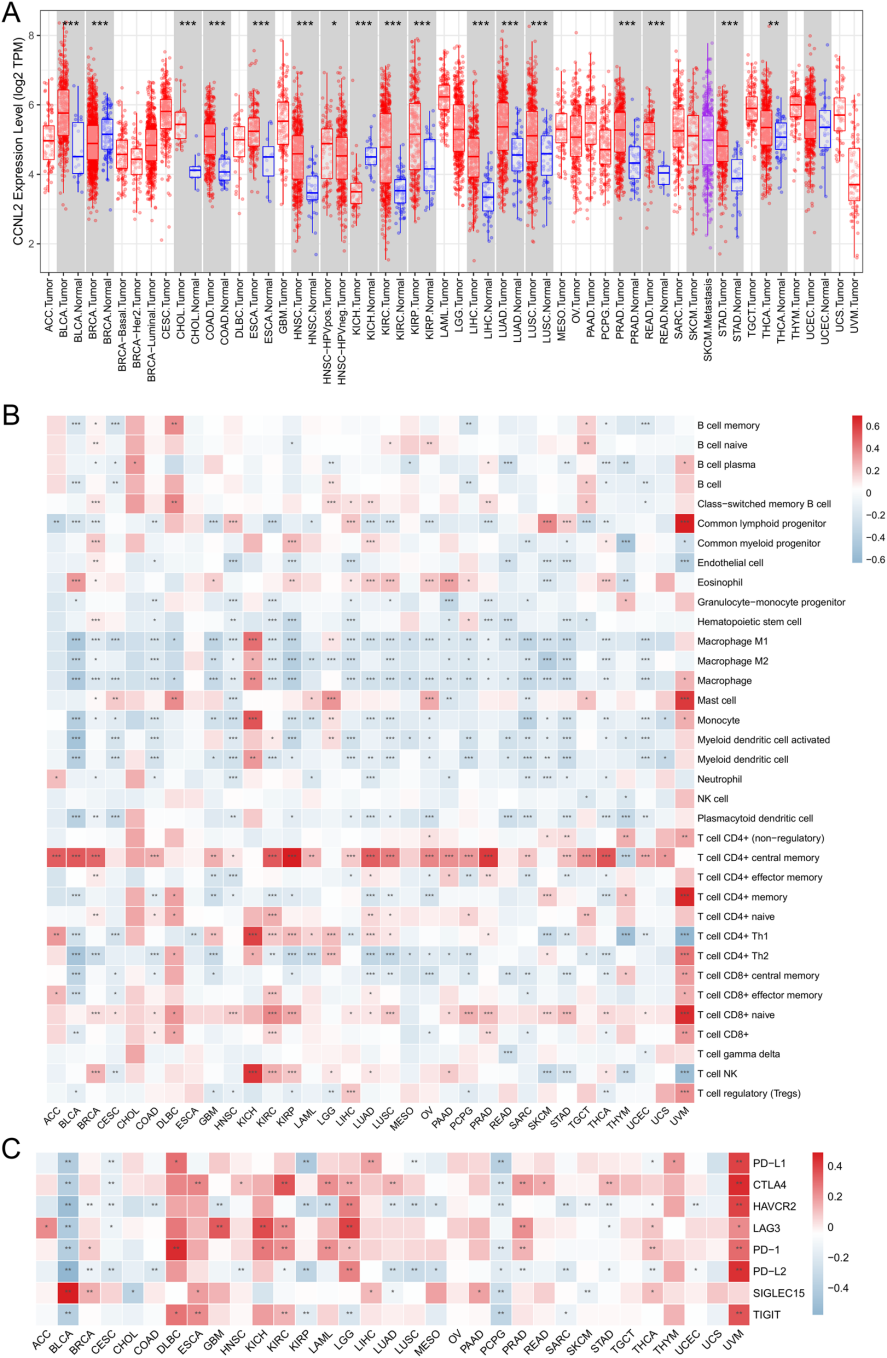
Pan-cancer analysis of CCNL2: **A** The expression of CCNL2 in pan-cancer analyzed by the TIMER database. **B** Association between the expression of CCNL2 and the immune infiltration levels in 33 cancer types. **C** Association between the expression of CCNL2 and the expression of immune checkpoint genes in 33 cancer types. (*p < 0.05, ** p < 0.01, *** p < 0.001)

Furthermore, differential expression of CCNL2 was observed in multiple cancers (Fig. 9A). Moreover, there was a strong correlation between CCNL2 and immune cells and immune checkpoint genes (Fig. 9B–C). The results of survival analysis showed that CCNL2 expression was related to the overall survival of BLCA, HNSC, KIRC, LGG and SARC patients and the progression-free survival of LGG and PRAD patients (Additional file 1: Figure S13).

## Discussion

Prostate cancer is the leading non-cutaneous cancer in men worldwide. The treatment of prostate cancer varies among different clinical states. In general, patients begin with localized disease, followed by the non-castrate rising PSA state and the non-castrate metastatic state. Finally, there are castration-resistant states which means a poor prognosis [34]. Despite the long-term survival in localized prostate cancer, castration-resistant prostate cancer remains largely incurable. The lethality of advanced disease is driven by the lack of therapeutic options that can generate durable responses [35]. In a previous study, functional interactions in ceRNA networks coordinated many biological processes and contributed to disease pathogenesis, such as human cancers [36]. To the best of our knowledge, the ceRNA network in prostate cancer is still inadequate. Therefore, we constructed ceRNA networks using the “GDCRNATools” package and obtained a ceRNA network (AC005154.6/hsa-miR-29c-3p/CCNL2), which provides insights for further exploring novel biomarkers for early diagnosis and treatment decisions in PCa.

Indeed, AC005154.6, hsa-miR-29c-3p and CCNL2 have all been explored for their possible roles in cancer. AC005154.6 was found to be a risk factor for patients with colorectal cancer [37]. Moreover, downregulation of hsa-miR-29c-3p was related to a poor prognosis in patients with laryngeal squamous cell carcinoma [38], non-small cell lung cancer [39] and bladder cancer [40]. CCNL2 is a novel member of the cyclin gene family. Studies have shown that it acts as a tumor suppressor protein in gastric cancer [41] and lung cancer [42]. This may also be validated in our pan-cancer analysis results. Although the p values were not less than 0.05, the HRs for STAD and LUAD were less than 1, indicating that high expression of CCNL2 might be a protective factor for these patients. In our study, AC005154.6 and CCNL2 expression were significantly higher in PRAD, and high expression of AC005154.6 and CCNL2 corresponded to poor prognosis. However, in contrast to AC005154.6 and CCNL2, hsa-miR-29c-3p expression was downregulated in PRAD, and the low expression level of hsa-miR-29c-3p indicated worse PFS in PRAD Fig. 9.

DNA methylation is an epigenetic mechanism which is mediated by DNA methyltransferases [43]. Tumors often exhibit abnormal patterns of cytosine DNA methylation in CpG dinucleotides, which contribute to disease progression. In prostate cancer, a high grade-specific difference in DNA methylation has been observed in gene promoters, gene bodies, and gene 3' ends [44]. In this study, we found that DNA methyltransferases were highly expressed in the CCNL2^high^ group. Furthermore, we found that in PCa tissues, CCNL2 methylation was significantly decreased, which might indicate that the upregulation of CCNL2 was due to low DNA methylation.

Recent studies have revealed that prostate cancer initiation and progression are extensively influenced by the tumor microenvironment [45, 46]. There is a reciprocal relationship between malignant tumor cells and the cells in the microenvironment [47]. In this study, we discovered that CCNL2 gene copy numbers were associated with the infiltration levels of most immune cells in prostate cancer and that CCNL2 expression had a notable correlation with CD8 + T cells. These findings suggested that the AC005154.6/hsa-miR-29c-3p/CCNL2 axis may influence the progression of PRAD via the tumor immune microenvironment.

There is persuasive evidence that the androgen-signaling axis plays an essential role in the pathogenesis of Pca [48]. Multiple cellular events of PCa are regulated by AR, including proliferation, migration, and differentiation. [49]. For the most part, tumors respond to ADT, but most become resistant to therapy within two years [50]. However, therapeutic regimens are poor when prostate cancer progresses to CRPC. In the last few years, immunotherapy, especially immune checkpoint inhibitors, has been widely used in clinical practice. The survival benefit in some patients with durable responses is astonishing. However, the benefit for the majority of patients is far from what we expect. Therefore, novel biomarkers are urgently needed for a better judgment of whether the application of immunotherapy is appropriate. In this study, we evaluated the association between immune checkpoint genes and the ceRNA network. Higher expression of CCNL2 corresponds to higher expression of CTLA4 and PD-1. Moreover, high CCNL2 expression was associated with a higher MSI score in patients. Through the GDSC database, patients with high expression of CCNL2 were not sensitive to bicalutamide. All the results suggested the possibility that immunotherapy rather than androgen deprivation therapy may benefit patients with high expression of CCNL2. Interestingly, we observed that patients in the CCNL2^high^ group were more sensitive to docetaxel than those in the CCNL2^low^ group. It became the standard of care for mCRPC in 2004 when docetaxel was the first systemic therapy to demonstrate a survival benefit [51]. The latest guidelines from the EAU still recommend it as a first-line treatment for mCRPC [52]. Considering that patients with high expression of CCNL2 exhibit more aggressive clinicopathological features, it seems reasonable that these patients had a higher sensitivity to docetaxel. Additionally, we applied the CellMiner and CMap databases to identify potential drugs to treat PCa.

Although we established a ceRNA network in PRAD, which appears to provide potential biomarkers to choose a suitable treatment regimen, several limitations also exist. First, animal experiments could further validate the function and mechanism of the AC005154.6/hsa-miR-29c-3p/CCNL2 axis in PARD. Moreover, the samples of tumor and adjacent normal tissues used for western blotting are limited.

In conclusion, we constructed a ceRNA network (AC005154.6/hsa-miR-29c-3p/CCNL2) that was related to the prognosis and treatment of PRAD. Moreover, a prognostic model was established based on the ceRNA network, and the AC005154.6/hsa-miR-29c-3p/CCNL2 axis provided guidance for the diagnosis, targeted therapy, and immunotherapy of prostate cancer.

## Supplementary Information

**Additional file 1: Figure S1.** Progression-free survival for the DEmiRNAsrelated to prognosis in PRAD. **Figure S2**: Progression-free survival for the DEmRNAsrelated to prognosis in PRAD. **Figure S3**: Progression-free survival for the DElncRNAsrelated to prognosis in PRAD. **Figure S4**: A correlation heatmap which exhibits the correlation among AC005154.6, hsa-miR-29c-3p and CCNL2. **Figure S5**: Progression-free survival of high-and low-risk groups in PRAD: (A) the cut-off obtained by the “survminer” package. (B) the Kaplan-Meier survival curve of high-and low-risk groups. **Figure S6**: Time dependent ROC curve analysis for survival prediction by the riskscore. **Figure S7**: (A)Univariate Cox regression analysis of correlations between risk score for PFS and clinical parameters(B)Multivariate Cox regression analysis of correlations between risk score for PFS and clinical parameters. **Figure S8**: The proportions of primary outcome of CR/PR and SD/PD in high-and low-risk groups. **Figure S9**: (A) Association between CCNL2 expression and sample type, nodal metastasis status in UALCAN. (*** p < 0.001, **** p < 0.0001). **Figure S10**: Waterfall plot displays the mutation status of genes with high mutation frequencies in CCNL2highand CCNL2lowgroups. **Figure S11**: (A) The functions of CCNL2 and its most correlated genes from GeneMANIA (B-C) GSEA analysis between CCNL2highand CCNL2low groups. **Figure S12**: Association between CCNL2 gene copy number and immune cells infiltration level. **Figure S13**: (A) Association between the expression of CCNL2 and overall survival in 33 cancer types. (B) Association between the expression of CCNL2 and progression-free survival in 33 cancer types.

**Additional file 2**: Criteria for determining ceRNA networks.

**Additional file 3: Table S1.** siRNA sequence.

**Additional file 4.** Sixty-one ceRNA networks constructed by identified DEmRNAs, DEmiRNAs, and DElncRNAs.

**Additional file 5.** Thirteen ceRNA networks correlated with patient survival.

**Additional file 6.** Prognosis model constructed by AC005154.6/hsa-miR-29c-3p/CCNL2 axis.

**Additional file 7.** Drugs significantly associated with the expression of CCNL2.

**Additional file 8.** Twenty-nine small molecules identified from CMap.

## Data Availability

Links of the websites used in this study are provided in supplementary materials, further inquiries can be directed to the corresponding author.
