# Supplementary material for: Integrated analysis to identify the AC005154.6/hsa-miR-29c-3p/CCNL2 axis as a novel prognostic biomarker associated with immune infiltration in prostate cancer
Source: Cancer Cell Int. 2022 Nov 11;22:346. doi: 10.1186/s12935-022-02779-5 (PMC9652791; doi:10.1186/s12935-022-02779-5)
Supplement: Supplementary file 1 — Additional file 1: Figure S1. Progression-free survival for the DEmiRNAsrelated to prognosis in PRAD. Figure S2: Progression-free survival for the DEmRNAsrelated to prognosis in PRAD. Figure S3: Progression-free survival for the DElncRNAsrelated to prognosis in PRAD. Figure S4: A correlation heatmap which exhibits the correlation among AC005154.6, hsa-miR-29c-3p and CCNL2. Figure S5: Progression-free survival of high-and low-risk groups in PRAD: (A) the cut-off obtained by the “survminer” package. (B) the Kaplan-Meier survival curve of high-and low-risk groups. Figure S6: Time dependent ROC curve analysis for survival prediction by the riskscore. Figure S7: (A)Univariate Cox regression analysis of correlations between risk score for PFS and clinical parameters(B)Multivariate Cox regression analysis of correlations between risk score for PFS and clinical parameters. Figure S8: The proportions of primary outcome of CR/PR and SD/PD in high-and low-risk groups. Figure S9: (A) Association between CCNL2 expression and sample type, nodal metastasis status in UALCAN. (*** p < 0.001, **** p < 0.0001). Figure S10: Waterfall plot displays the mutation status of genes with high mutation frequencies in CCNL2highand CCNL2lowgroups. Figure S11: (A) The functions of CCNL2 and its most correlated genes from GeneMANIA (B-C) GSEA analysis between CCNL2highand CCNL2low groups. Figure S12: Association between CCNL2 gene copy number and immune cells infiltration level. Figure S13: (A) Association between the expression of CCNL2 and overall survival in 33 cancer types. (B) Association between the expression of CCNL2 and progression-free survival in 33 cancer types. [file 12935_2022_2779_MOESM1_ESM.pdf]

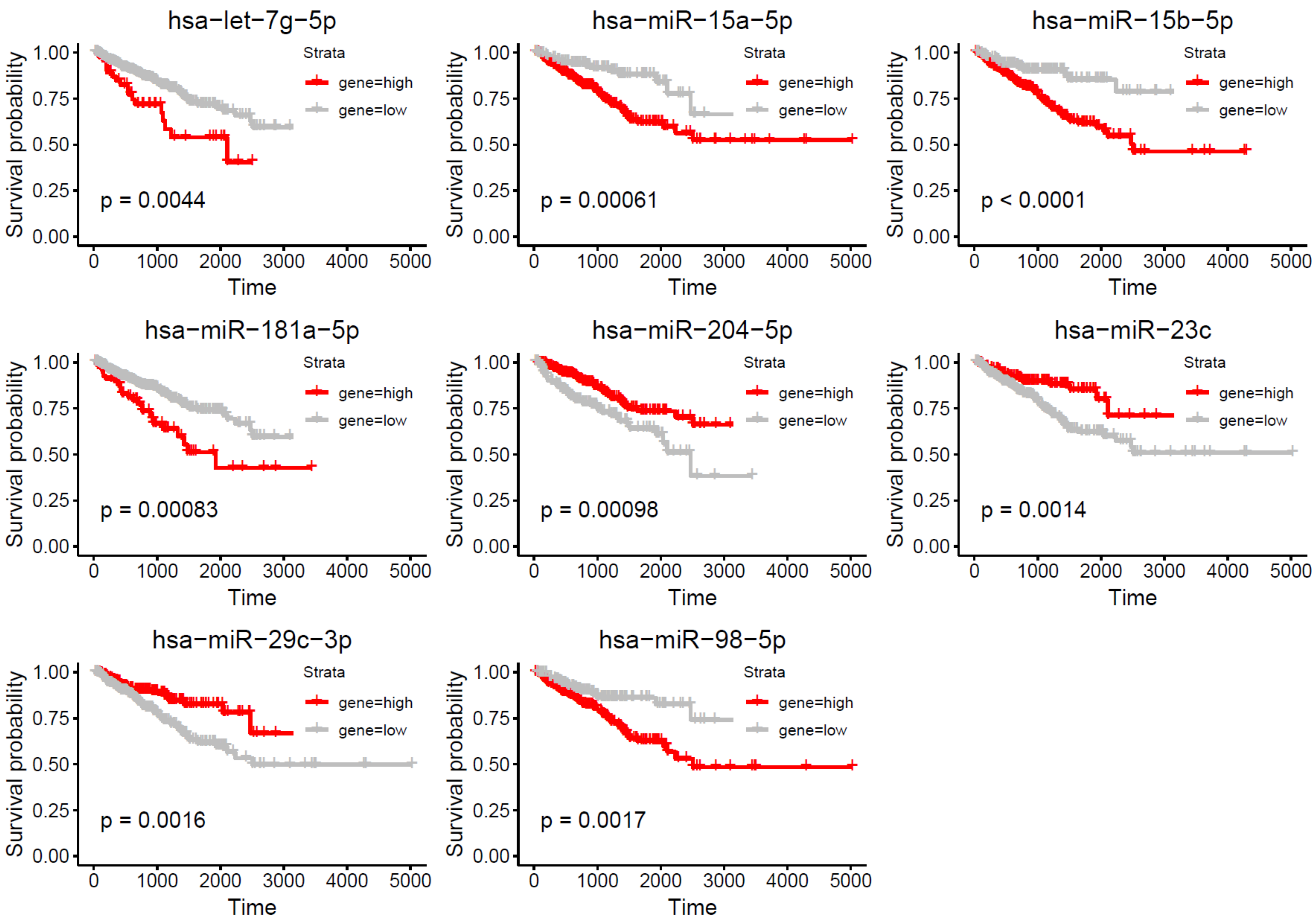

Figure S1: Progression-free survival for the DEmiRNAs related to prognosis in PRAD

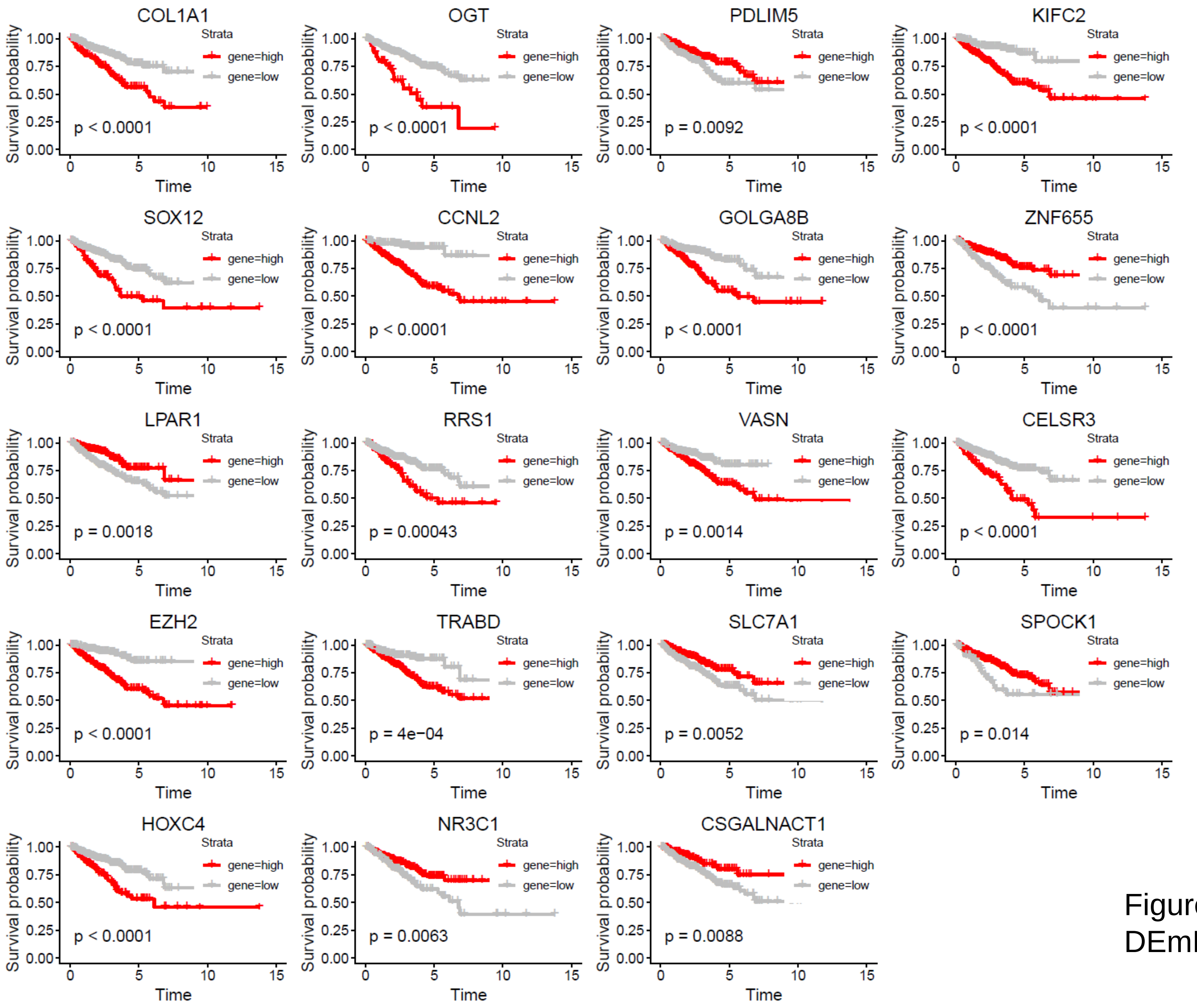

Figure S2: Progression-free survival for the DEmRNAs related to prognosis in PRAD

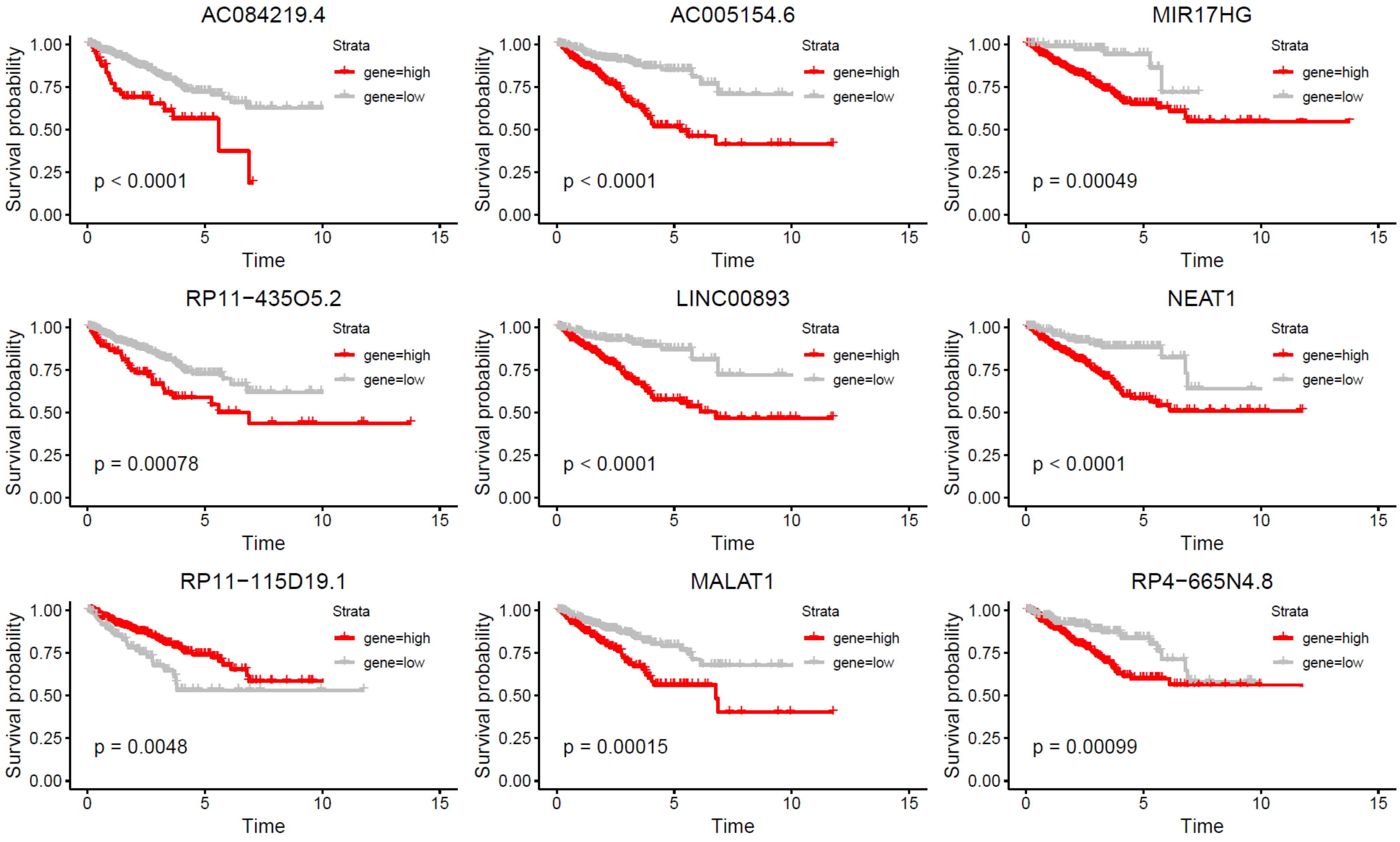

Figure S3: Progression-free survival for the DElncRNAs related to prognosis in PRAD

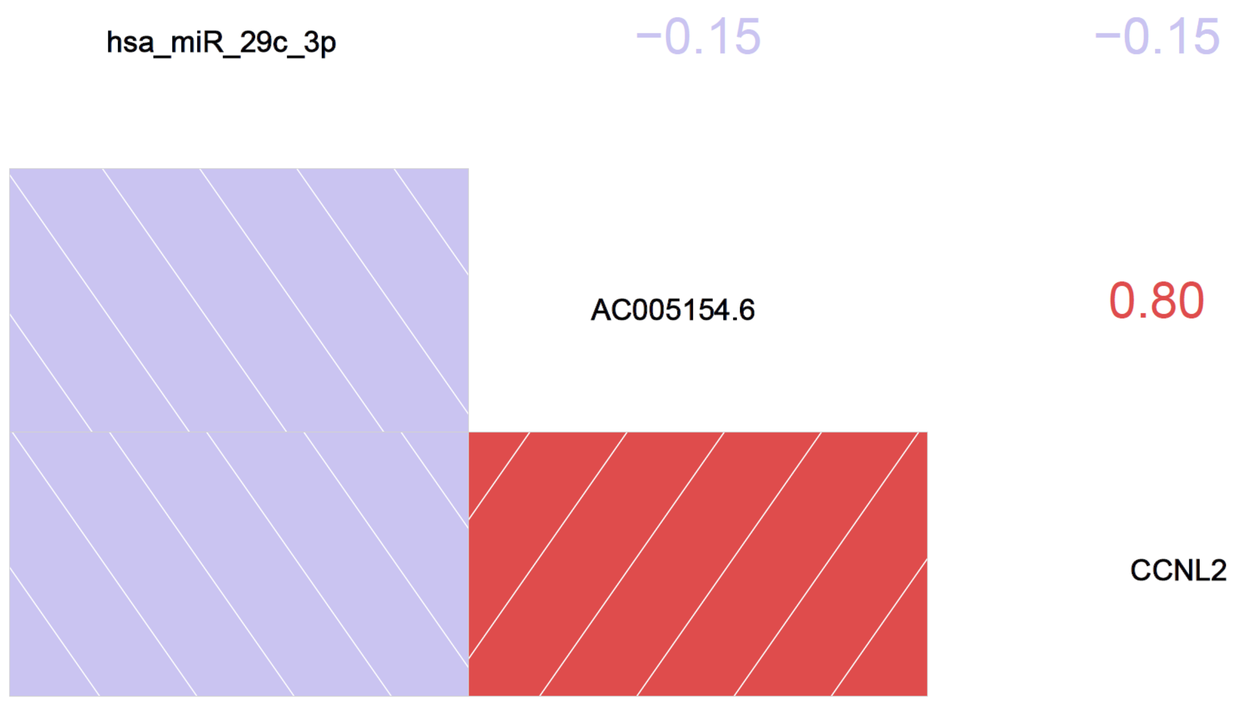

Figure S4: A correlation heatmap which exhibits the correlation among AC005154.6, hsa-miR-29c-3p and CCNL2

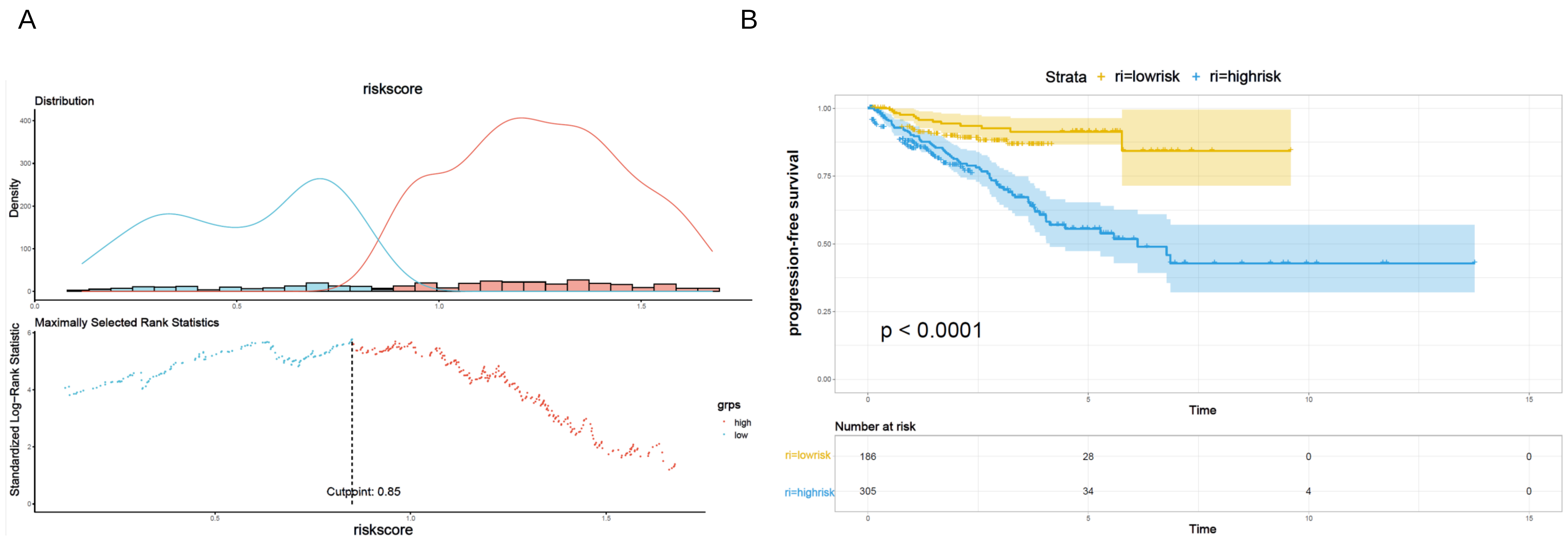

Figure S5: Progression-free survival of high- and low-risk groups in PRAD: (A) the cut-off obtained by the "survminer" package. (B) the Kaplan-Meier survival curve of high- and low-risk groups.

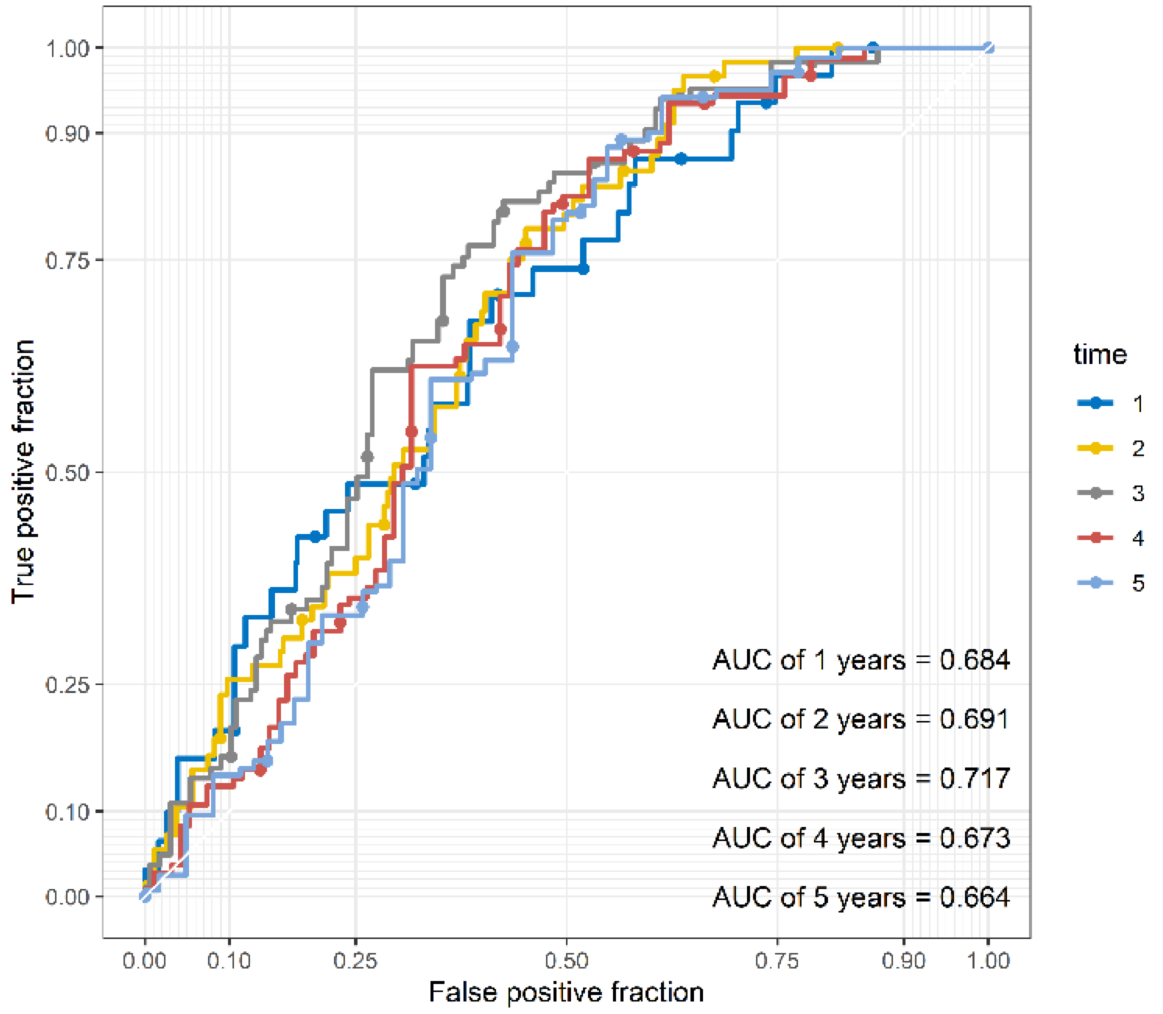

Figure S6: Time dependent ROC curve analysis for survival prediction by the riskscore.

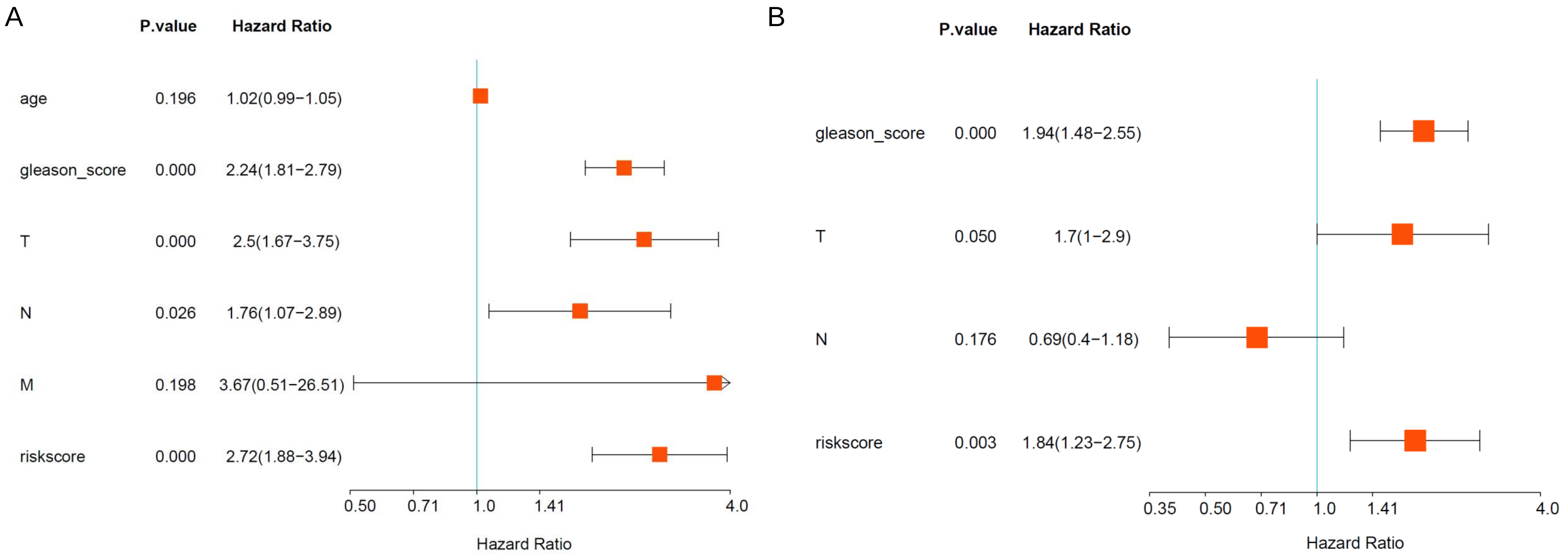

Figure S7: (A)Univariate Cox regression analysis of correlations between risk score for PFS and clinical parameters(B)Multivariate Cox regression analysis of correlations between risk score for PFS and clinical parameters

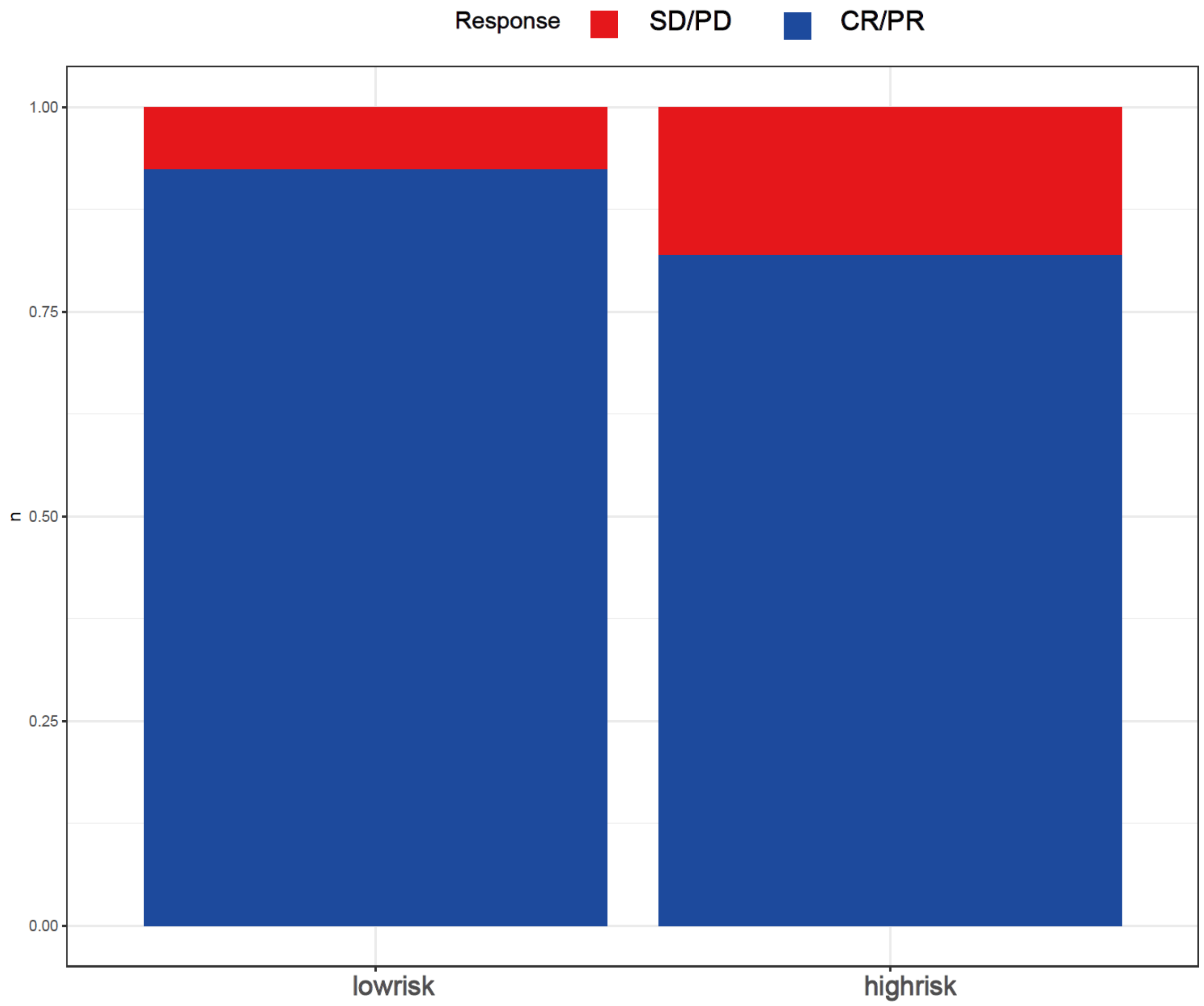

Figure S8: The proportions of primary outcome of CR/PR and SD/PD in high- and low-risk groups

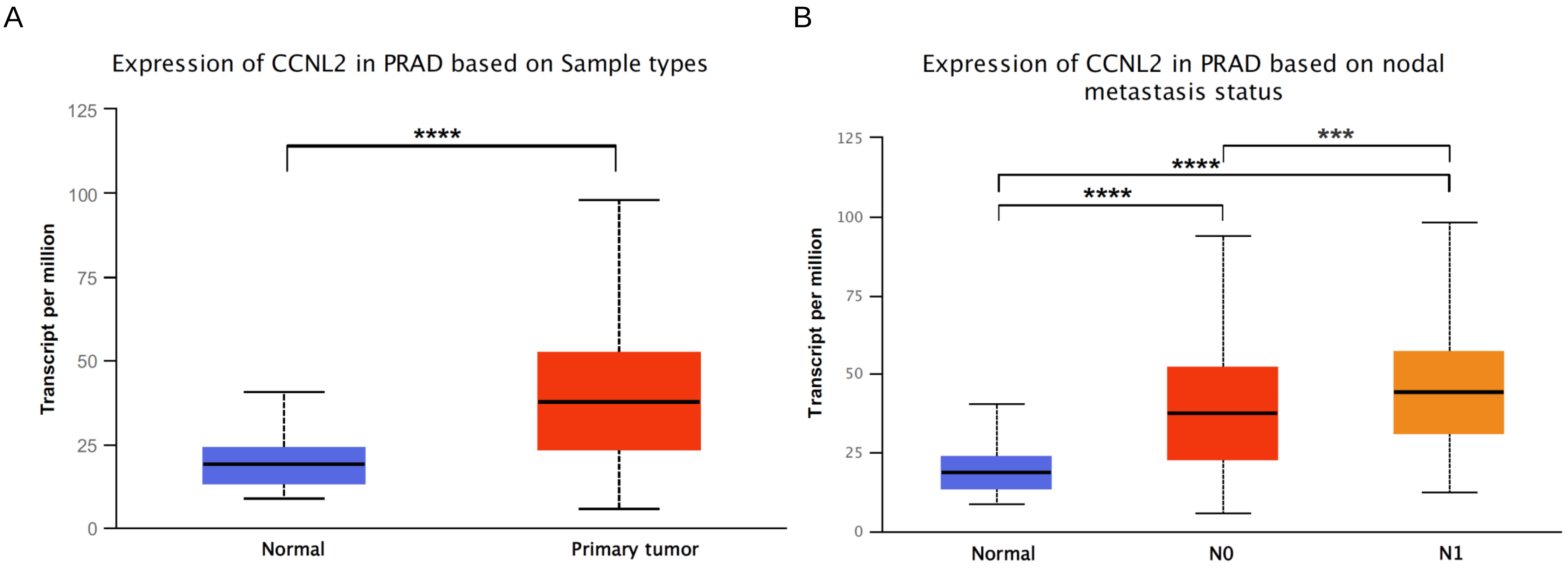

Figure S9: (A) Association between CCNL2 expression and sample type, nodal metastasis status in UALCAN. (*** $p < 0.001$, **** $p < 0.0001$)

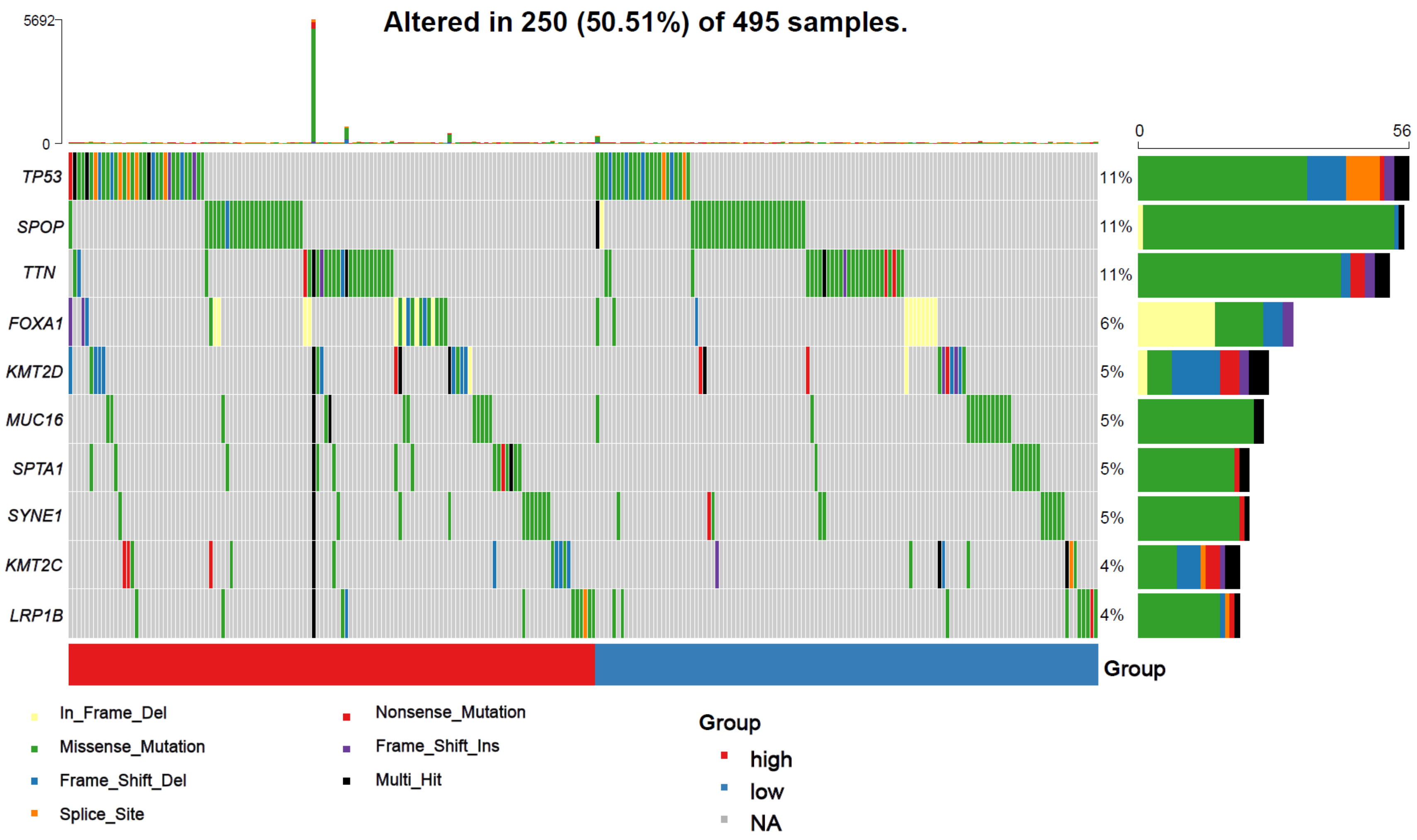

Figure S10: Waterfall plot displays the mutation status of genes with high mutation frequencies in CCNL2^{high} and CCNL2^{low} groups.

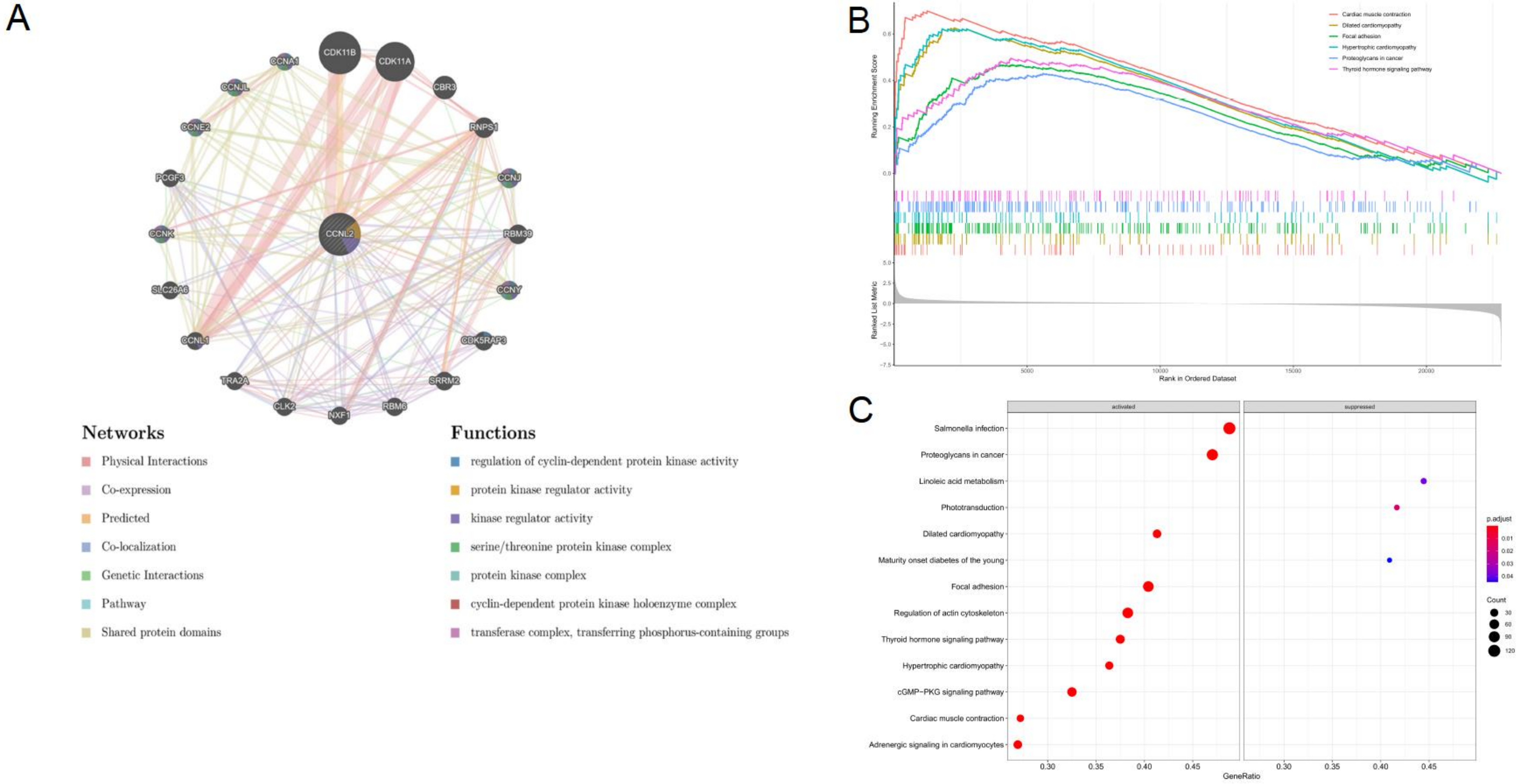

Figure S11: (A) The functions of CCNL2 and its most correlated genes from GeneMANIA (B-C) GSEA analysis between $CCNL2^{high}$ and $CCNL2^{low}$ groups.

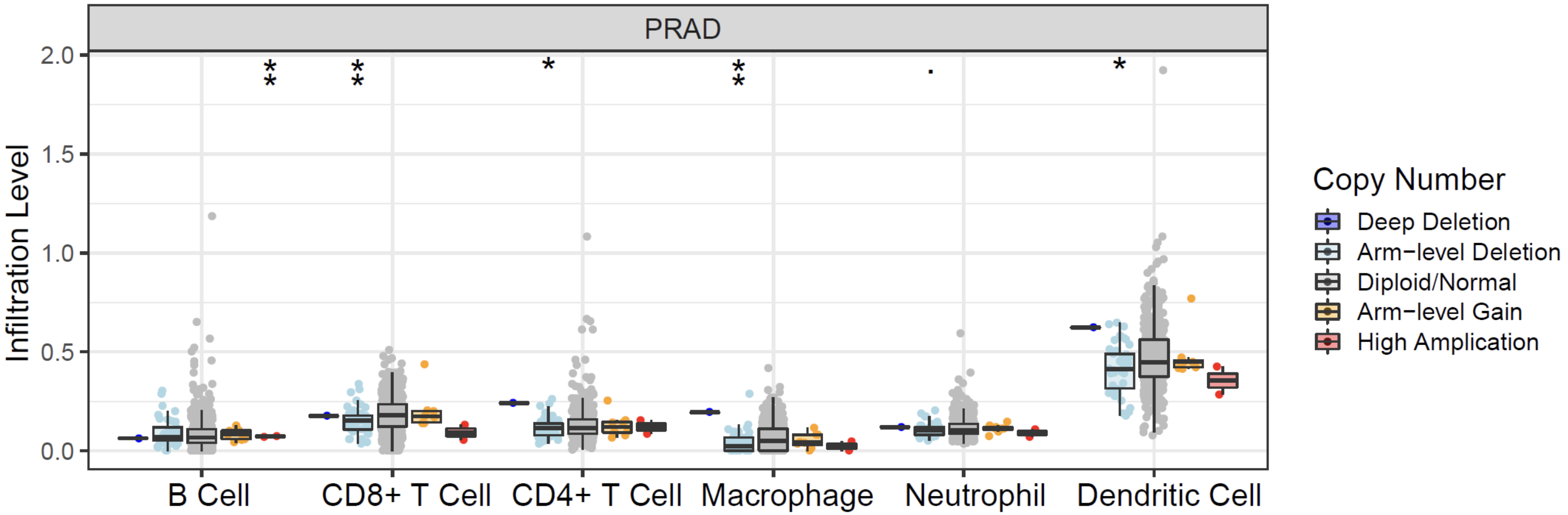

Figure S12: Association between CCNL2 gene copy number and immune cells infiltration level.

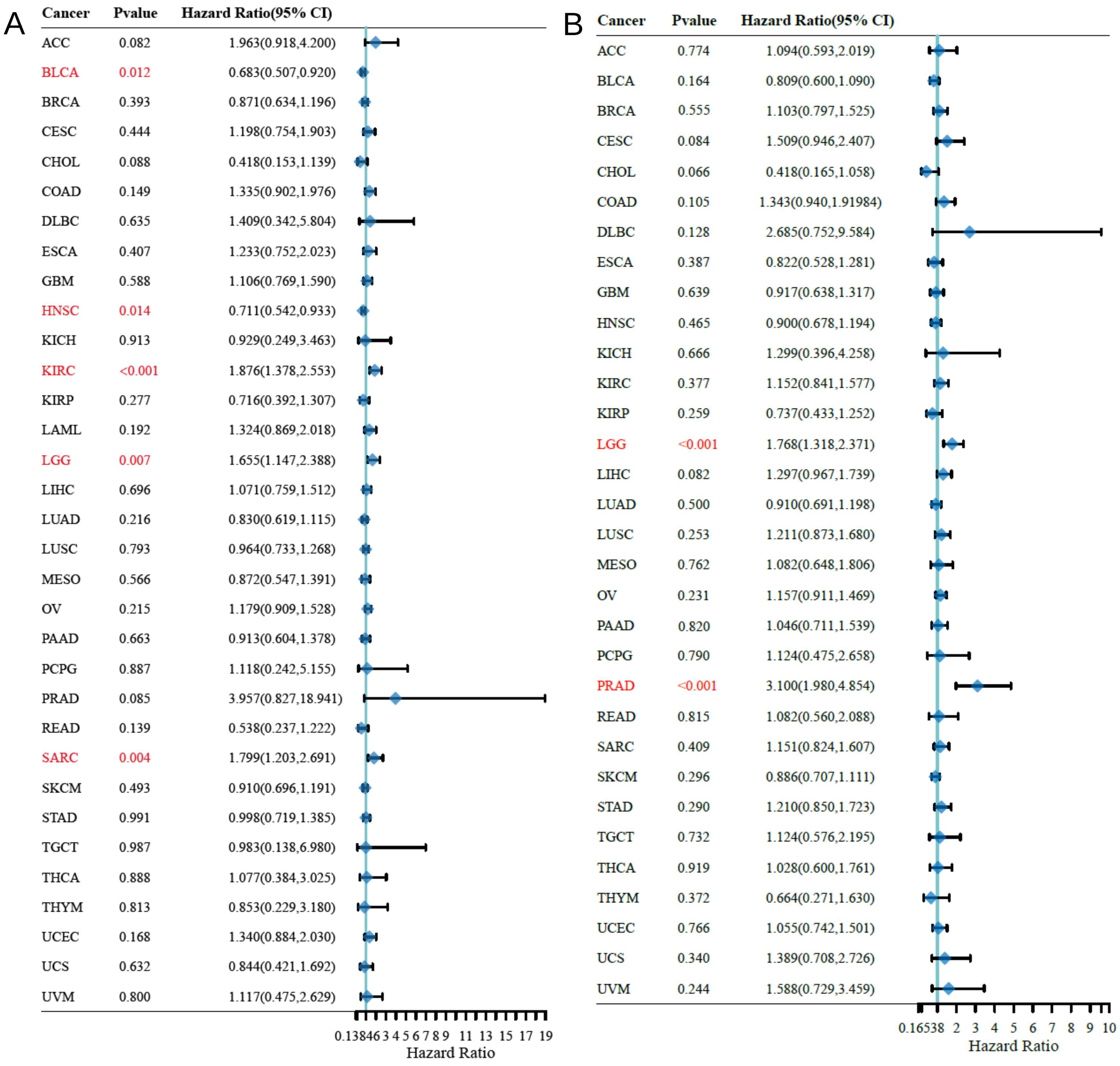

Figure S13: (A) Association between the expression of CCNL2 and overall survival in 33 cancer types. (B) Association between the expression of CCNL2 and progression-free survival in 33 cancer types.
