# Supplementary material for: Integrated analysis to identify the AC005154.6/hsa-miR-29c-3p/CCNL2 axis as a novel prognostic biomarker associated with immune infiltration in prostate cancer
Source: Cancer Cell Int. 2022 Nov 11;22:346. doi: 10.1186/s12935-022-02779-5 (PMC9652791; doi:10.1186/s12935-022-02779-5)
Supplement: Supplementary file 2 — Additional file 2: Criteria for determining ceRNA networks. [file 12935_2022_2779_MOESM2_ESM.docx]

**Criteria for determining ceRNA networks:**

Three criteria were used for “GDCRNATools” package to determine the competing endogenous interactions:

(1) the lncRNA and mRNA must share significant number of miRNAs

(2) expression of lncRNA and mRNA must be positively correlated

(3) those common miRNAs should play similar roles in regulating the expression of lncRNA and mRNA.

**Abbreviations**

ADT: androgen deprivation therapy

AR: androgen receptor

CRPC: castration-resistant prostate cancer

ceRNA: competitive endogenous RNA

DElncRNAs: Differentially expressed lncRNAs

DEmiRNAs: Differentially expressed miRNAs

DEmRNAs: Differentially expressed mRNAs

PFS: progression-free survival

FDR: false discovery rate

GEO: Gene Expression Omnibus

GSEA: Gene Set Enrichment Analysis

GO: Gene Ontology

HR: hazard ratio

HPA: Human Protein Atlas

KEGG: Kyoto Encyclopedia of Genes and Genomes

MSI: Microsatellite Instability

mCRPC: metastatic castration-resistant prostate cancer

OS: overall survival

PCa: prostate cancer

PRAD: prostate cancer

siRNA: small interfering RNA

TCGA: The Cancer Genome Atlas

**Links of website used in this article:**

The Cancer Genome Atlas database: <https://portal.gdc.cancer.gov/>

Gene Expression Omnibus database: <https://www.ncbi.nlm.nih.gov/geo/>

starBase v2.0: <http://starbase.sysu.edu.cn/starbase2/index.php>

TargetScan: <http://www.targetscan.org/vert_71/>

TIMER: <https://cistrome.shinyapps.io/timer/>

TIMER 2.0: <http://timer.cistrome.org/>

HPA database: <https://www.proteinatlas.org/>

UALCAN: <http://ualcan.path.uab.edu/>

DiseaseMeth version 2.0: <http://bio-bigdata.hrbmu.edu.cn/diseasemeth/>

MEXPRESS: <https://mexpress.be/>
