# Supplementary material for: Integrated analysis to identify the AC005154.6/hsa-miR-29c-3p/CCNL2 axis as a novel prognostic biomarker associated with immune infiltration in prostate cancer
Source: Cancer Cell Int. 2022 Nov 11;22:346. doi: 10.1186/s12935-022-02779-5 (PMC9652791; doi:10.1186/s12935-022-02779-5)
Supplement: Supplementary file 3 — Additional file 3: Table S1. siRNA sequence. [file 12935_2022_2779_MOESM3_ESM.docx]

**Table S1: siRNA Sequence**

| siRNA name | Target sequence |
| --- | --- |
| CCNL2si-1 | GCATACGGGACGTCATCAA |
| CCNL2si-2 | GGAAAGACGAGTTCTCAAA |
